# Categorisation of Mobile EEG: A Researcher's Perspective

**DOI:** 10.1155/2017/5496196

**Published:** 2017-12-04

**Authors:** Anthony D. Bateson, Heidi A. Baseler, Kevin S. Paulson, Fayyaz Ahmed, Aziz U. R. Asghar

**Affiliations:** ^1^School of Engineering, University of Hull, Hull HU6 7RX, UK; ^2^Hull York Medical School, University of Hull, Hull HU6 7RX, UK; ^3^Department of Psychology, University of York, York YO10 5DD, UK; ^4^Department of Neurology, Hull Royal Infirmary, Hull HU3 2JZ, UK

## Abstract

Researchers are increasingly attempting to undertake electroencephalography (EEG) recordings in novel environments and contexts outside of the traditional static laboratory setting. The term “mobile EEG,” although commonly used to describe many of these undertakings, is ambiguous, since it attempts to encompass a wide range of EEG device mobility, participant mobility, and system specifications used across investigations. To provide quantitative parameters for “mobile EEG,” we developed a Categorisation of Mobile EEG (CoME) scheme based upon scoring of device mobility (*D*, from 0, off-body, to 5, head-mounted with no additional equipment), participant mobility (*P*, from 0, static, to 5, unconstrained running), system specification (*S*, from 4, lowest, to 20, highest), and number of channels (*C*) used. The CoME scheme was applied to twenty-nine published mobile EEG studies. Device mobility scores ranged from 0*D* to 4*D*, participant mobility scores from 0*P* to 4*P*, and system specification scores from 6*S* to 17*S*. The format of the scores for the four parameters is given, for example, as (2*D*, 4*P*, 17*S*, 32*C*) and readily enables comparisons across studies. Our CoME scheme enables researchers to quantify the degree of device mobility, participant mobility, and system specification used in their “mobile EEG” investigations in a standardised way.

## 1. Introduction

Mobile or ambulatory EEG is an increasingly active area of research utilised in a variety of applications and scenarios such as outdoor urban environments [1, 2], sports activities [3], and brain-computer interfacing [4–6]. Typically, participant movement during EEG recordings is discouraged in order to reduce data artifacts. Mobile EEG seeks to obtain recordings whilst movement is taking place, but its success has been impeded by low system specification and the mounting position of the EEG equipment. EEG systems are now becoming available, which lend themselves to mobile applications, although in general they have lower system specifications when compared to static EEG systems.

Mobile EEG approaches include walking on a treadmill whilst being tethered to immobile EEG equipment [7, 8], walking outdoors with a wireless mobile EEG headset combined with a rucksack mounted PC [9], and being moved on a trolley whilst wearing a virtual reality headset to provide the sensation of movement [10]. These diverse approaches cause ambiguity because they are all termed “mobile EEG” by researchers and yet exhibit wide variation in EEG device mobility, participant mobility, and system specification.

One technique that has been used to address the problem of misleading terminology in biomedical research and the differing interpretation of some terms across practitioners is categorisation based upon scales for parameters of interest [11, 12]. An advantage of using a categorisation scheme is that it standardises scores so they can be exchanged between practitioners with greater clarity. Since the term “mobile EEG” lacks this form of standardisation, a categorisation scheme that encompasses the range of mobility of EEG equipment, mobility of the participant, and the main features of EEG system technical specification would provide researchers with a standardised way of quantifying “mobile EEG” as used in studies.

A categorisation scheme for mobile EEG with sufficient measure and usability would allow researchers to determine the potential of specific EEG systems (and related equipment) and to guide development of new and more mobile experimental protocols. By extension, it would also allow EEG system developers to include in the design of systems attributes that are of importance to the research community as technology advances become possible. The literature contains a number of EEG system comparison studies where usability [13, 14], signal quality [15], performance [16], and electrode types [17] were compared. However, no categorisation schemes are currently available for “mobile EEG.”

In the current investigation, we first reviewed twenty-nine published research investigations selected because they either had “mobile EEG” (or “ambulatory EEG”) in the paper title and/or involved some form of participant mobility whilst EEG recordings were being acquired. We extracted the key features related to equipment used, equipment mounting position, participant activity, and EEG system specification. We next developed a novel categorisation scheme for “mobile EEG” based upon scoring the following key parameters: device mobility, participant mobility, and system specification. The specific parameter score descriptors for device mobility and participant mobility were derived from descriptions given in the twenty-nine “mobile EEG” published research investigations and one static EEG study. The categorisation scheme was then applied retrospectively to the thirty published studies, and a subset of these was taken to illustrate the range of unique categorisation scores in the parameters covered by the developed scoring scheme.

## 2. Development of the Categorisation of Mobile EEG (CoME) Scheme

To develop descriptors for device mobility, participant mobility, and system specification, we reviewed thirty published research studies. More specifically, we selected studies that either had “mobile EEG” (or ambulatory EEG) in the title and/or involved some form of participant mobility whilst EEG recordings were being taken. This enabled a range of each parameter to be derived from these studies, along with informative descriptors for each score. We selected one investigation that used EEG in a static setting [20] to provide contrast for the “mobile EEG” studies and allow us to benchmark appropriate scales. This study was particularly suitable as an example of static EEG since participant movement was actively discouraged via training provided to participants prior to recording.

The studies included are shown in Table 1 with details of the year of publication, equipment mounting positions, EEG system used, and participant activity during EEG recordings. This information was used to derive both the device mobility parameter scores and those of the participant mobility scores. When information was either missing from the publication or ambiguously described, this has been reported in the table.

### 2.1. Device Mobility

**Figure 1:**
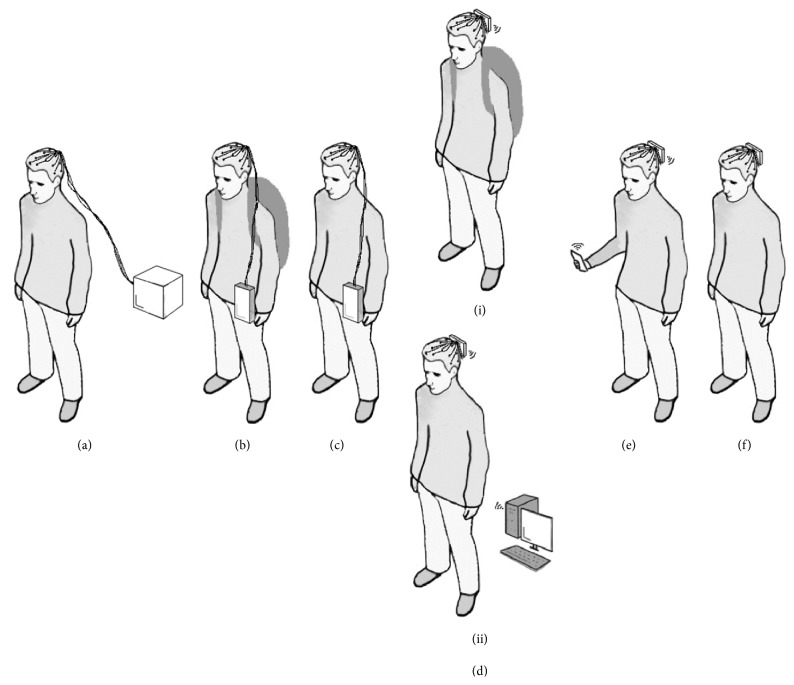
Various mounting positions of the EEG device and associated equipment on the participant. (a) All equipment is off-body mounted and participant tethered via cabling to EEG acquisition equipment. (b) Waist-mounted (or back-mounted) with additional equipment located in a rucksack. (c) All equipment is waist-mounted. (d) Head-mounted EEG system, with additional equipment located (i) in a rucksack or (ii) off-body tethering participant via limited-range wireless link. (e) Head-mounted and requires smartphone/tablet. (f) Head-mounted. Acquisition, storage, and analysis equipment is integrated within the headset.

The device mobility score reflects the mounting position (off-body, waist-mounted, or head-mounted), along with the level of physical restriction placed upon the participant by the EEG acquisition system.  Figure 1 shows examples of the various mounting positions of the device and associated equipment on the participant.  Table 2 provides the device mobility scores and descriptors. The mounting modalities taken from the published studies (see Table 1) fit to the descriptors for scores of 0 to 4. The descriptor for score of 5 is aspirational and is taking a logical projection of what could be developed from the descriptor for score of 4.

When all of the equipment is off-body mounted and the participant is tethered to the equipment via cabling, it is clear that the equipment is static and therefore is scored as 0. When the EEG amplifier is mounted on the waist (or the back) of a participant, movement-related artifacts are likely because of electrode displacement [9, 40]. Leads cannot be fastened to the participant sufficiently well to completely remove electrode wire movement as this will then cause restricted head movement. This coupled with the length of electrode wires can result in increased electromagnetic interference [41]. This provides scores of 1 and 2, where the difference between these is the restriction placed upon the participant. If additional equipment is placed in a rucksack on the participant, this scores 1 as the participant is encumbered with this additional load and in turn increases the weight to be carried along with susceptibility to system movement artifacts [42]. When no additional equipment is used, a higher of score of 2 is used to reflect that the participant is not encumbered with an additional load.

With EEG amplifiers that are completely head-mounted, movement-related artifacts are reduced and head movements are not restricted [9]. However, in general, the headset needs to wirelessly link to another piece of equipment such as a PC (see Table 1). This applies restrictions on the participant which take the form of equipment placed in a rucksack and therefore encumbering with an additional load or the additional equipment is stored off-body and the participant is constrained by the wireless connection range. Both of these modalities score 3 as in both conditions participants are constrained in some way. When the equipment is replaced with a smartphone, which is inherently mobile, the participant is clearly far less constrained [21, 33] and this condition scores 4. The next logical step is a modality where not even a smartphone is required and this scores 5. Although no example of such a system has been found, it has been included, as future EEG system developments could well achieve this level.

### 2.2. Participant Mobility

The scaling descriptors for participant mobility were based upon the activities described in the published EEG studies (see Table 1).  Table 3 gives the score associated with each descriptor. This score reflects the level of participant mobility in the context of instructions given by the researcher to participants during a study. It is not unusual for participants to perform more than one type of activity, and in such cases we scored the activity involving the highest participant mobility. From the list of studies, activities ranged from static to treadmill running. The scores captured this range from 0 to 4 with a score of 5 allowing for future work where participants run unconstrained or are playing sport.

When movement is discouraged by researchers, this is done in order to decrease the likelihood of movement-related artifacts [25]. In protocols where participants were lying, sitting, or standing still, a score of 0 was applied as the participants are static and not mobile. In protocols where participants were lying, sitting, or standing with localised movement (e.g., finger tapping or button pressing), a score of 1 was given to recognise the introduction of movement, albeit localised. These localised movements are defined as occurring without actual displacement of the whole body.

Treadmill walking, although constraining the participant in terms of direction and pace of movement, is a further increase upon localised movement and this type of activity scores 2. Indoor or outdoor unconstrained walking scores 3 as the participant is not constrained in terms of direction and pace, as is the case with a treadmill. The aspects of environment such as indoor/outdoor or urban/rural whilst having a sensory impact on the participant [1, 9] do not have a quantifiable impact on their mobility and are therefore not considered in the scoring or descriptors.

With treadmill running being the greatest level of mobility found in any of the studies in Table 1, a score of 4 was given to this level of activity. The same score was also given for a study where the participants had to carry packages of different sizes and weights (0.5 to 15 Kg) whilst walking [2] and a study of epileptic outpatient data where stair climbing was assumed to be the most mobile activity [18]. The justification for scoring the two disparate activities the same is that they both include activities that are more than just walking. This allows a score of 5 to be applied to unconstrained running or sport. No study was found to include such a level of mobility but this score allows for this to be captured in the future.

### 2.3. System Specification

EEG system specification is an important consideration in mobile EEG research studies. A system that is considered to be highly mobile may only have a low system specification that adversely affects signal quality. Conversely, a system that is considered to be static (off-body mounted) typically has a higher system specification. Therefore, a system specification score was developed, in addition to the device and participant mobility scores.  Table 4 lists the EEG systems used in previously published studies (see Table 1) along with the sampling rate, bit resolution, number of channels, battery life, and electrode type. These values were used to formulate scores to differentiate between differing system specifications.

The system specification score consists of four attributes added together, with each attribute ranging from 1 to 5. It was decided not to start the system specification scale for each parameter from 0 as the interpretation of this would be unclear, and even the most basic device would have some utility. It is also important to note that the impact of a device encumbering the participant has been incorporated into the device mobility score rather than the system specification score since they are factors that affect participant mobility.

Since the emphasis is on mobility, the electrode type has been captured as this impacts the likelihood and severity of motion artifacts. A specific example is that of dry electrodes that are much more difficult to secure to the participant and movement in relation to the participant's body occurs more readily [43]. Gel-based electrodes provide improved signal quality in comparison to saline [9]. The EEG systems used in Table 4 can be broken down into either dry-, saline-, or gel-based electrodes that are either passive or active and unshielded or shielded. The scoring for this was 1 to 3 for the dry, wet, and gel (or cream), respectively, with the addition of one score each if the electrodes are active and shielded (see Table 5). This provides a score range from 1 to 5; dry, passive, and unshielded give a score of 1, and gel, active, and shielded a score of 5.

Since the bit resolution impacts upon the accuracy of the data recorded and the sampling rate of the system governs the temporal resolution, we included both these parameters in the system specification scoring. These are also attributes that are usually reported by researchers. It should be noted that the sampling rates recorded from the EEG systems used in our thirty selected studies (see Table 1) fall into sequences of either 125, 250, 500,… or 128, 256, 512,… because of the underlying technology used. It was therefore decided that 125/128, 250/256, 500/512,… would score the same as their temporal resolutions are very similar.

The reporting of the system's specification in published studies does not always include the attributes required to be captured by our proposed scoring system. In these cases, system manufacturer specification details were sought to supplement the missing information. Where systems have been developed and therefore manufacturer specification details do not exist, a range of possible scores is reported to encompass the potential variation in specification score.

Battery life is an important consideration of mobile EEG. For an EEG system to be fully mobile, it has to be battery-powered, and the charge life of the battery directly governs the period of active monitoring that can take place [44]. A battery life attribute was included in the system specification score so duration of use could be captured, and where equipment was not battery-powered, this was also reported.

A rating scale of 1 to 5 for each system specification attribute was used. The range for each attribute is from the lowest used in commercially available systems up to the highest and beyond to cover future expected technological developments. The score assigned for each attribute of the system specification is dictated by the actual bit resolution, sampling rate, battery life, and electrode type used in the research investigation.  Table 6 shows the system attributes and scoring of the system specification.

Scores for bit resolution, sampling rate, battery life, and electrode type were added together to form a single combined total score for system specification (*S*) out of a maximum possible score of 20. For example, a bit resolution of 14, sampling rate of 512 Hz, a battery life of 8 hours, and active shielded gel electrodes would give a total system specification score of 1 + 3 + 2 + 5 = 11*S*.

### 2.4. Number of Channels

To develop a scoring scale for the number of channels used in an EEG study, many factors would have to be considered which relate to the specific type of investigation being undertaken. The type of analysis to be performed quite often necessitates a certain number of channels for validity, such as distributed source reconstruction [45] and spatial filtering methods [46]. Although, in general, the range of possible analysis approaches systematically increases with spatial densities and therefore a scale could be created on such a basis, the positioning of the electrodes presents another aspect of the problem. A steady-state visual evoked potentials experiment using electrodes only located posteriorly is different from an imagined motor activity experiment that uses electrodes localised anteriorly. Therefore, in our categorisation scheme, we opted to report the number of channels (*C*) separately, for example, 32*C*.

## 3. Application of the Categorisation of Mobile EEG (CoME) Scheme

**Figure 2:**
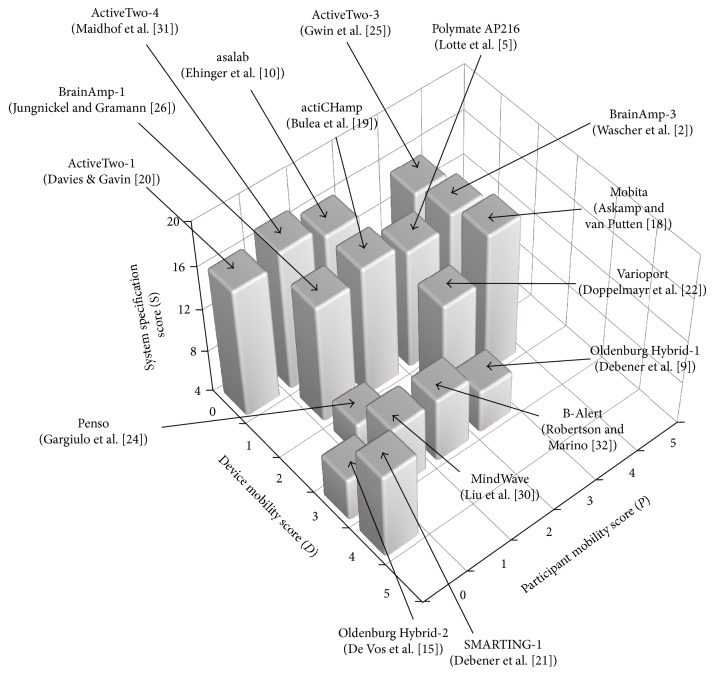
3D plot showing the device and participant mobility scores and the system specification scores for each selected research investigation and associated EEG systems. Refer to Table 8 for the number of channels used in each selected study.

We applied our developed Categorisation of Mobile EEG (CoME) scheme to all thirty published research studies listed in Table 1. The specification score (*S*) for each EEG system (bit resolution, sampling rate, battery life, and electrode type) is presented in Table 7. The scores for device mobility (*D*), participant mobility (*P*), total score for system specification (*S*), and number of channels (*C*) are presented in Table 8.  Figure 2 presents in a 3D plot the *D*, *P*, and *S* scores for a selected subset of the thirty studies (comprising sixteen studies), which illustrate the range of specific scores obtained using our CoME scheme. For each of the selected studies, we summarize below the EEG equipment and participant activity, the resultant *D*, *P*, and *S* scores, number of channels used, and the final categorisation score:

### 3.1. ActiveTwo-1 (BioSemi, Netherlands)

Davies and Gavin [20] used an off-body mounted system in which participants were seated and therefore were static. Consequently, device mobility (*D*) and participant mobility (*P*) were ranked at the lowest point of each scale (0*D* and 0*P*). This study used a BioSemi ActiveTwo EEG system with recordings made using 24-bit sampling resolution (score = 4), at a sampling rate of 1024 Hz (score = 4) and battery life of 10 hours (score = 3) and electrodes were active, shielded, and gel-based (score = 5), yielding a system score of 16*S*. Since 32 channels were used, the total score for the BioSemi ActiveTwo system as used in this study was (0*D*, 0*P*, 16*S*, 32*C*).

### 3.2. ActiveTwo-3 (BioSemi, Netherlands)

The study by Gwin et al. [25] also used a BioSemi ActiveTwo system but involved participants walking and running on a treadmill with the EEG acquisition equipment mounted off-body on a rack above the treadmill. This configuration yields a device mobility score of 0*D*. The treadmill running of the participant scored 4*P*. The system score is 15*S* which is different from Davies and Gavin [20] because of the lower sampling rate of 512 Hz (score = 3). However, the number of channels used was greater at 248 channels. The overall score is (0*D*, 4*P*, 15*S*, 248*C*).

### 3.3. ActiveTwo-4 (BioSemi, Netherlands)

Motion capture was used in conjunction with EEG whilst participants played a digital piano in a study by Maidhof et al. [31]. A BioSemi ActiveTwo system was used but this time with a sampling rate of 8192 Hz (score = 5), which combines with the bit resolution, battery life, and electrode type to form a score of 17*S*. The device was mounted off-body and scores 0*D*. The participants were seated and were performing localised movement which scores as 1*P*. The number of channels used was 32, making the overall score (0*D*, 1*P*, 17*S*, 32*C*).

### 3.4. actiCHamp (Brain Vision, USA)

User-driven treadmill walking was an investigation undertaken by Bulea et al. [19]. A figure shows participants wearing an EEG cap that is then wired to one of several back-mounted pieces of equipment. This arrangement has been scored as 1*D*, and because the study used treadmill walking, the score for participant mobility was 2*P*. The EEG system consisted of 24-bit sampling (score = 4), 500 Hz sampling frequency (score = 3), and battery life of 24 hours (score = 4) summed to form a score of 16*S*. 64 channels of EEG were used for this study, providing a combined score of (1*D*, 2*P*, 16*S*, 64*C*).

### 3.5. asalab (ANT Neuro, Netherlands)

Ehinger et al. [10] used the term “mobile EEG study” to describe participants moving with a trolley. The trolley was used to mount all of the equipment in a static EEG system format. Since the device was mounted off-body (on the trolley), it was given a score of 0*D* for device mobility. As part of the study, participants moved by pushing the trolley within guide rails, and a score of 2*P* for participant mobility was given. The EEG equipment system specification was 24-bit resolution (score = 4), with a 1024 Hz sampling rate (score = 4) and battery life of 10 hours (score = 3), and active, shielded, gel electrodes were used (score = 5), combining to give 16*S*. Since 128 EEG channels were used, the overall score was (0*D*, 2*P*, 16*S*, 128*C*).

### 3.6. B-Alert (Advanced Brain Monitoring, USA)

Monitoring responses in the prefrontal cortex (PFC) and motor cortex (MC) during cycling-based exercise was the purpose of the investigation by Robertson and Marino [32]. They used a B-Alert mobile EEG system to capture the data; since this is a head-mounted system that connects to a PC via a wireless connection, it was given a score of 3*D*. The participants were seated on fixed exercise cycles during the study which scored 2*P*. The B-Alert system specification, as used, consisted of a 16-bit sampling resolution (score = 2), 256 Hz sampling rate (score = 2), and battery life of 8 hours (score = 2), and passive shielded conductive cream-based electrodes were used (score = 4), combining to give 10*S*. Overall score including the 20 channels' score was (3*D*, 2*P*, 10*S*, 20*C*).

### 3.7. BrainAmp-1 (Brain Products, Germany)

Participants stood in front of a projection screen and had to point, in a study by Jungnickel and Gramann [26]. The EEG system is placed in a backpack and consequently scores 1*D*. The participants in this study were standing still and pointing, which is a localised movement and scores 1*P*. The BrainAmp system specification, as used, consisted of a 16-bit sampling resolution (score = 2), 500 Hz sampling rate (score = 3), and battery life of 30 hours (score = 5), and active, shielded, gel-based electrodes were used (score = 5), combining to give 15*S*. The overall score including the 156 channels' score was (1*D*, 1*P*, 15*S*, 156*C*).

### 3.8. BrainAmp-3 (Brain Products, Germany)

Wascher et al. [2] also used a BrainAmp EEG system in a study that recorded EEG whilst participants carried packages of various weights (0.5 to 15 Kg) whilst walking. The EEG equipment was mounted on the participants in a belt bag located at their lower back and scored 1*D*. A score of 4*P* was attributed to the activity carried out by participants since it went beyond unconstrained walking with the inclusion of package carrying. The system score is 16*S*, which is different from Jungnickel and Gramann [26], because of the higher sampling rate of 1000 Hz (score = 4). The combination of scores provides an overall score of (1*D*, 4*P*, 16*S*, 28*C*).

### 3.9. MindWave (NeuroSky, USA)

Participants were seated during a driving simulation study undertaken by Liu et al. [30]. They used a head-mounted MindWave system that requires a wirelessly linked PC to process and store the EEG data. The device mobility was scored as 3*D* as the laptop/PC restricts the range of the participants. Since the participants were undertaking a seated driving simulation task in which small amounts of localised physical movement were involved, it was scored as 1*P*. The MindWave system has a 16-bit sampling resolution (score = 2), a 500 Hz sampling rate (score = 3), and battery life of 10 hours (score = 3) with a dry, passive, shielded electrode (score = 2) to give a combined score of 10*S*. The overall score for this study with a single channel used was (3*D*, 1*P*, 10*S*, 1*C*).

### 3.10. Mobita (TMSi, Netherlands)

The Mobita EEG system was evaluated by Dutch neurologists in a study by Askamp and van Putten [18]. The device was waist-mounted and did not require any additional equipment and thus scored 2*D*. Since participants in the study were outpatients, it was assumed that everyday home activities would be the type of activities undertaken. Stair climbing was therefore considered the most mobile of activities within a home environment and so scored 4*P*. The EEG system provided a 24-bit sampling resolution (score = 4), a 2000 Hz sampling rate (score = 5), and battery life of 19 hours (score = 4) with gel-based, passive, shielded electrodes (score = 4) to give a combined score of 17*S*. The overall score for this study with 32 EEG channels used was (2*D*, 4*P*, 17*S*, 32*C*).

### 3.11. Oldenburg Hybrid-1 (Modified Emotiv, USA)

Debener et al. [9] undertook a study in which participants walked outside unconstrained. This investigation used what is referred to as the Oldenburg system, comprised of the data acquisition electronics from an EPOC system fitted to an electrode cap (Easycap, Germany). This modified system was developed in an attempt to increase data quality by improving electrode connection to the participant's scalp. However, the resulting data recordings are still limited by the comparatively low system specification of the acquisition electronics. The device mobility was scored as 3*D* since the EEG acquisition equipment was in a rucksack mounted on the participant. This study scored relatively high for participant mobility (3*P*) as participants were walking outside (constrained only by the weight of the rucksack-housed laptop). The acquisition electronics are from an EPOC EEG system with a 14-bit resolution (score = 1), sampling rate of 128 Hz (score = 1), and battery life of 6 hours (score = 2) with the upgrade of electrodes to being gel-based, passive, and shielded (score = 4), giving the score of 8*S*. The system was still limited to 14 channels and our categorisation gave an overall score of (3*D*, 3*P*, 8*S*, 14*C*).

### 3.12. Oldenburg Hybrid-2 (Modified Emotiv, USA)

De Vos et al. [15] compared the performance of the Oldenburg Hybrid to that of a traditional amplifier in a seated BCI speller task. Although the participants were not required to carry additional equipment in a rucksack, they were still limited by the range of the wireless link to a corresponding PC and therefore scored 3*D*. With the participants being seated whilst performing a visual ERP speller task, they were seated without moving and gain a score of 0*P*. The acquisition electronics and electrode types remain the same as with Debener et al. [9] and therefore score 8*S*. The system again has 14 channels with the categorisation score becoming (3*D*, 0*P*, 8*S*, 14*C*).

### 3.13. Penso (Noncommercial)

Gargiulo et al. [24] studied seated participants performing imagined motor activity. They built their own EEG system that was waist-mounted and scored 2*D*. Since the participants were seated and physical movement was limited to eyes opening and closing and button pressing, a score of 1*P* was assigned. Their EEG system provided them with 16-bit sampling resolution (score = 2) and a 256 Hz sampling rate (score = 2). The battery life was not mentioned in the publication and since it is not a commercial product, commercial documentation could not be used. A score of 1 was given, and along with electrodes that were dry, passive, and shielded (score = 2), this gave a score for the system specification of 7*S*, and with 8 EEG channels, the overall score was (2*D*, 1*P*, 7*S*, 8*C*).

### 3.14. Polymate AP216 (TEAC Corp., Japan)

Lotte et al. [5] studied EEG recordings from participants performing corridor walking. The EEG system and a laptop were stored in a backpack which the participants wore and scored 1*D*. Since the participants were walking in an unconstrained manner, this was scored as 3*P*. The Polymate EEG system provided them with 16-bit sampling resolution (score = 2), a 1000 Hz sampling rate (score = 4), battery life of 18 hours (score = 4), and active, shielded, gel-based electrodes (score = 5) summed to give 15*S*. 3 channels were used during this study and contribute to the overall score of (1*D*, 3*P*, 15*S*, 3*C*).

### 3.15. SMARTING-1 (mBrainTrain, Belgrade, Serbia)

Debener et al. [21] used a mobile EEG system with participants seated indoors. The emphasis was on the unobtrusive flexible printed electrodes they had used which were located around the ears. EEG recordings were made using SMARTING, which is a head-mounted system that transmits data wirelessly to a smartphone or PC. Since a smartphone was used in their study, the device mobility scored 4*D*. The participants' movement was essentially static whilst seated indoors when recordings took place and scored 0*P*. The SMARTING EEG system provided the researchers with samples at 24-bit sampling resolution (score = 4), a sampling rate of 500 Hz (score = 3), battery life of 5 hours (score = 2), and gel-based, passive, and unshielded electrodes (score = 3), giving 12*S*. The overall score with 16 EEG channels used was (4*D*, 0*P*, 12*S*, 16*C*).

### 3.16. Varioport (Becker Meditec, Germany)

Doppelmayr et al. [22] performed static EEG recordings during rest periods in ultralong running events. They also performed EEG recordings with participants walking slowly with eyes closed (hand-led by a member of support crew), and it is this part of the study which was scored since it contained the most physical movement. EEG recordings were made using a light weight, waist-mounted system called Varioport which scored 2*D*. The participants' movement involved periods of slow walking and scored 3*P*. The Varioport EEG system provided the researchers with samples at 16-bit sampling resolution (score = 2), a sampling rate of 2000 Hz (score = 5), and battery life of 96 hours (score = 5). No information regarding the type of electrodes used in the study is supplied in the publication, and attempts made to find this missing information via manufacturer documentation have also failed to help. A score was applied by taking the only information available (passive) and applying the lowest scores for the unknown parameters (score = 3). A total score of 13*S* is given but this could be higher with a score range of 13–17*S*. With Varioport being used to provide 10 channels, the overall score was (2*D*, 3*P*, 13–17*S*, 10*C*).

## 4. Discussion

In the current investigation, we have reviewed thirty published research investigations from which we have developed a novel categorisation scheme for “mobile EEG” based upon scores of the parameters, device mobility, participant mobility, and system specification, whilst also reporting the number of channels used. The parameter score descriptors were derived following review of thirty published research investigations. The categorisation scheme was then applied retrospectively to all thirty published studies and a subset of these (sixteen studies) was taken to illustrate the range of specific categorisation scores in the parameters covered by the developed scoring system.

The results show a broad range in the scores for device mobility, participant mobility, and system specification. Our results highlight the need for such a categorisation scheme to be adopted and utilised by researchers, as it provides a quantitative and more accurate description than the vague term “mobile EEG.” In addition, the categorisation has been designed to provide the scores in a convenient format. This allows researchers to readily capture and compare the actual meaning of the term “mobile EEG” in the context of a specific researcher's study. We encourage researchers to apply our categorisation scheme to their own mobile EEG systems and research contexts when using the ambiguous term, “mobile EEG,” in their publications and reports. It should be noted that users of our categorisation scheme should not equate lower or higher scores with subjective judgements. Our intention is only to quantify the level of device mobility, participant mobility, system specification, and number of channels used in a comparable way across studies.

Based upon our categorisation scores of the thirty published studies, there may be an indication that EEG systems with higher system specification scores are associated with lower scores for device mobility. For example, the studies by Maidhof et al. [31] and Ehinger et al. [10] have higher device specification scores of 17*S* and 16*S*, respectively, but lower scores for device mobility of 0*D*. In contrast, EEG systems with lower scores for system specification scored higher for device mobility. For example, the studies by Debener et al. [9] and Stopczynski et al. [33, 39] scored 8*S* and 6*S*, respectively, for system specification but scored 3*D* and 4*D*, respectively, for device mobility. Our developed CoME scheme could enable researchers, in future investigations using a larger number of included studies, to determine whether there are any significant relationships between device mobility, participant mobility and system specification.

Our categorisation scoring scheme will aid in the development of new mobile EEG systems, by both research and commercial communities, by making it possible to clearly identify quantifiable improvements and to clarify reporting of “mobile EEG” in publications. Fully head-mounted EEG systems are especially appropriate for recordings to be acquired in mobile settings. Combining such a head-mounted system with a smartphone would give high scores for device and participant mobility.

Our categorisation scheme is capable of informing investigators of the potential for increasing device and participant mobility and thereby highlighting future research possibilities. For example, in the study by Gargiulo et al. [24], the imagined motor activity could be undertaken whilst participants walk, as the device mobility score of 2*D*, in principle, allows a higher level of mobility to be introduced. In the studies by Stopczynski et al. [33, 39], using the EPOC system, device mobility scores were 4*D*, but since the investigation involved participants sitting still, the participants' mobility scores were limited to 0*P*. This indicates that there is a potential for additional investigations to be designed in which participants' mobility could be introduced or increased. A further example is in the studies by Zink et al. [38] and Debener et al. [21] who used the SMARTING system. If Zink et al. [38] were to use a smartphone instead of a PC, as in the study by Debener et al. [21], to record the transmitted data, then the study score for device mobility would increase from 3*D* to 4*D* to enable novel protocols.

Another usage of our categorisation scheme by researchers could be in determining the degree of device mobility, participant mobility, system specification, and number of channels used within and between research investigations. To better facilitate this process, we have included a Categorisation of Mobile EEG (CoME) form in the Supplementary Material available online at https://doi.org/10.1155/2017/5496196. It is hoped by including this easy-to-use resource that researchers will be encouraged to quantify their research in terms of the categories covered. From the perspective of our categorisation scheme, an ideal “mobile EEG” system would score a maximum of (5*D*, 5*P*, 20*S*) and represents a fully head-mounted system that does not require additional equipment for data recording. The participant would be able to undertake extreme activities such as unconstrained running, and the system specification would be greater than 24-bit resolution and greater than 1000 Hz sampling with a battery life of more than 24 hours. We could find no study and EEG system combination with this level of device and participant mobility coupled with this level of system specification. Our expectation is that, given the pace of current developments, the higher scores for system and device/participant mobility descriptors are likely to be achievable in the foreseeable future.

There are two ways in which the CoME scheme can be applied: (1) retrospectively and (2) prospectively. We applied it retrospectively to thirty published studies and scored according to what the research reported. There is usually a reason why researchers have not maximised the settings. This could also relate to the equipment used as some systems are modular and, in order to use the equipment in a certain way, compromises may have been made. If the scheme is used to plan a study and therefore the scheme is to be applied prospectively, a system's maximum settings would be scored.

### 4.1. Limitations of the CoME Scheme

In the CoME scheme, the total system specification is a combined score reflecting the electrode type, bit resolution, sampling rate, and battery life. Our recommendation is that researchers consider both the total system specification score and the individual scores for these parameters. It is possible that the CoME system specification score is not fully reflective of a researchers' expected change in EEG signal quality within a certain research context. For instance, an improvement in systems specification when using active electrodes instead of passive ones may be considered by a researcher to represent an increase in signal quality, but, using the CoME scheme, there would only be a score change from 0 to 1. However, when gel-based electrodes with wires of minimal length and associated electronics are in close proximity (head-mounted configuration), using active electrodes may not produce an increase in signal quality and a CoME score allocation of one may be considered as high.

There are potentially a wide range of system specification parameters that we could have included such as impedance and wireless connection range but we opted to only include bit resolution, sampling rate, battery life, and electrode type in our categorisation scheme. We included these four attributes as they are usually reported in published EEG research studies or can be found with relative ease from manufacturers' documentation. Some publications do not report details of the EEG system used and corresponding settings. We recommend that research investigators, as part of good publication practice, report at least the following parameters: EEG system name, EEG system manufacturer (where appropriate), bit resolution, sampling rate, battery life, electrode type, and number of channels used.

For electrode impedance, published research studies typically only provide a general statement such as* “impedance was kept below 5 kΩ”* rather than giving a precise value for each channel and for each participant. In addition, impedance is a dynamic variable that changes throughout the course of a study and it is assumed that researchers will deal with this as a matter of course when obtaining research data fit for publication. Another reason for selecting the parameters chosen is that a scoring scale can be generated with increasing gradations; for example, increasing sampling rate gives a higher score. Such an increasing gradation scale is not possible to generate for different electrode metal types (tin versus silver versus gold), where the application rather than the level of mobility of participants is the main factor of consideration.

Although our categorisation scheme has been developed from the perspective of the researcher and has descriptors that cover EEG equipment mobility, participant mobility, and system specification, it does not consider the perspective of the participant. It is possible that the participant may have experienced, for example, some discomfort related to the EEG equipment that the researchers had been unaware of or that items of equipment were encumbering during the investigation. Participants could be asked to provide scores for comfort, weight, and aesthetic form and these could be added to our categorisation scheme. For example, the study by Hairston et al. [13], which utilised participant-reported comfort ratings for EEG systems (1 = very comfortable, 7 = very uncomfortable), could potentially be incorporated into our categorisation scheme. The subjectivity of comfort assessments would be a problem when developing a participant-focused scoring system using descriptors, particularly as participant assessments may change over time. The addition of several participant-focused parameters to our categorisation scheme would make the format less concise and comparisons more difficult. Perhaps a separate participant categorisation scheme could be developed to address these issues, but it would have to accurately capture and quantify participant perspectives, which are rarely sought.

## 5. Conclusions

In conclusion, we have reviewed thirty published research studies that use the term “mobile EEG” or contain participant mobility whilst EEG is being acquired. From this review, we developed a categorisation scheme for “mobile EEG” studies based upon scoring for device mobility, participant mobility, system specification, and number of channels used in order to remove the inherent ambiguity in the way this term is used by researchers. The results of applying our categorisation scheme retrospectively to a range of published researches shows that it captures the degree to which the EEG equipment is mobile, the degree of participants' movement in the study, the main attributes of the system specification which are readily available, and the number of channels used. The format of the resultant scores is concise and enables convenient comparison across different research studies. Our categorisation of EEG (CoME) form is intended to be a useful aid for researchers to conveniently categorise their mobile EEG studies as well as in the design and development of mobile EEG equipment (see the supplementary material).

## Supplementary Material

Categorisation of Mobile EEG (CoME) scoring form.

## Figures and Tables

**Table 1:** Mobile EEG studies.

Study	Year	EEG system	Equipment mounting position	Participant activity
Askamp and van Putten [18]	2014	Mobita	Waist-mounted	Epileptic outpatients, stair climbing
Aspinall et al. [1]	2015	EPOC	Head-mounted, wireless link to back-mounted laptop	Outside walking
Bulea et al. [19]	2014	actiCHamp	Back-mounted (according to figure in paper)	Treadmill walking
Castermans et al. [4]	2011	ANT	Presumed off-body but not stated	Treadmill walking
Davies and Gavin [20]	2007	ActiveTwo	Off-body	Seated watching & listening
Debener et al. [9]	2012	Oldenburg Hybrid	Head-mounted, wireless link to back-mounted laptop	Indoor and outdoor walking
Debener et al. [21]	2015	SMARTING	Head-mounted, wireless link to smart device	Seated indoors
De Vos et al. [15]	2014	Oldenburg Hybrid	Head-mounted, wireless link to PC	Seated screen speller task
Doppelmayr et al. [22]	2012	Varioport	Waist-mounted	Slow walking
Duvinage et al. [16]	2013	EPOC & ANT	Presumed off-body but not stated	Treadmill walking
Ehinger et al. [10]	2014	asalab	Off-body	Constrained walking with trolley
Fitzgerald et al. [23]	2013	Profusion	Waist-mounted	Epileptic outpatients sleep monitoring
Gargiulo et al. [24]	2008	Penso	Presumed waist-mounted but not stated	Seated, eyes open & closed, button pressing
Gramann et al. [7]	2010	ActiveTwo	Off-body	Treadmill walking
Gwin et al. [25]	2010	ActiveTwo	Off-body	Running on treadmill
Jungnickel and Gramann [26]	2016	BrainAmp	Rucksack-mounted, wireless link to PC	Standing and pointing
Klonovs et al. [27]	2013	EPOC	Head-mounted, wireless link to PC	Not made clear. Presume seated driving
Li et al. [28]	2014	Not specified	Off-body	Human centrifuge
Lin et al. [29]	2014	Cognionics	Head-mounted, wireless link to PC	SSVEP whilst treadmill walking
Liu et al. [30]	2013	MindWave	Head-mounted, wireless link to PC	Seated driving
Lotte et al. [5]	2009	Polymate AP216	Back-mounted EEG device and laptop	Corridor walking
Maidhof et al. [31]	2014	ActiveTwo	Off-body	Keyboard playing
Robertson and Marino [32]	2015	B-Alert	Head-mounted, wireless link to PC	Exercise cycling
Stopczynski et al. [33]	2014	EPOC	Head-mounted, wireless link to smart device	Seated imagined finger tapping
Wagner et al. [34]	2012	BrainAmp	Off-body	Robotic-assisted treadmill walking
Wang et al. [35]	2014	NuAmp	Head-mounted, wireless link to PC	VR-simulated driving whilst seated
Wascher et al. [2]	2014	BrainAmp	Back-mounted	Indoor physical box sorting task
Wong et al. [36]	2014	MindWave	Head-mounted, wireless link to PC	Screen-based shape tracing while seated
Zander et al. [37]	2017	V-Amp	Off-body	Seated driving
Zink et al. [38]	2016	SMARTING	Head-mounted, wireless link to back-mounted laptop	Cycling

**Table 2:** Device mobility scores.

Device mobility score (*D*)	Descriptor
0	All equipment off-body mounted and participant tethered via cabling to EEG acquisition equipment.
1	Waist-mounted (or back-mounted) with additional equipment located in a rucksack.
2	All equipment is waist-mounted.
3	Head-mounted EEG system, with additional equipment located in a rucksack or off-body.
4	Head-mounted and requires smartphone/tablet.
5	Head-mounted and does not require any additional equipment.

Note that the level of participant mobility is not taken into account when considering device mobility. For example, if a study used a head-mounted system that did not require a PC or smartphone and the participant was instructed by the researcher to remain as still as possible, the device would be scored as 5*D*.

**Table 3:** Participant mobility scores.

Participant mobility score (*P*)	Descriptor
0	Lying, sitting, or standing still.
1	Lying, sitting, or standing with localised movement, for example, finger tapping or button pressing.
2	Constrained walking/cycling.
3	Unconstrained walking/cycling.
4	Walking and carrying, climbing stairs, and constrained running.
5	Unconstrained running and vigorous physical exercise or sport.

**Table 4:** Specifications of EEG systems as used in published research studies.

EEG system	Sampling rate (Hz)	Bit res. (bits)	Number of channels	Battery life (hours)	Electrode type		
					Dry/saline/gel	Passive/active	Unshielded/shielded
ActiveTwo [7, 20, 25, 31]	512, 1024, 8192	24	32, 248	10	Gel	Active	Shielded
asalab [10]	1024	24	128	10	Gel	Active	Shielded
B-Alert [32]	256	16	20	8	Conductive cream	Passive	Shielded
EPOC [1, 16, 27, 33]	128	14	14	6	Saline	Passive	Not stated
MindWave [30, 36]	500	16	1	10	Dry	Passive	Shielded
Mobita [18]	2000	24	32	19	Gel	Passive	Shielded
Oldenburg Hybrid [9, 15]	128	14	14	6	Gel	Passive	Shielded
Penso [24]	256	16	8	Not stated	Dry	Passive	Shielded
Profusion [23]	512	16	32	15	Gel	Passive	Not stated
SMARTING [21, 38]	500	24	16, 24	5	Gel	Passive	Not stated
Varioport [22]	2000	16	10	4 days	Not stated	Passive	Not stated
ANT [4, 16]	512, 2048	24	32, 128	5	Gel	Passive	Shielded
Polymate AP216 [5]	1000	16	3	18	Gel	Active	Shielded
V-Amp [37]	2000	24	16	N/A USB	Dry	Active	Shielded
BrainAmp [2, 26, 34]	500, 1000, 2500	16	28, 120, 156	30	Gel	Active	Shielded
NuAmp [35]	500	22	32	N/A USB	Gel	Passive	Unshielded
actiCHamp [19]	500	24	64	24	Gel	Active	Shielded
Cognionics [29]	250	24	10 out of 32	8	Dry	Active	Shielded

**Table 5:** Electrode type scoring.

Passive (0) Active (1)	Unshielded (0) Shielded (1)	Dry (1)
		Wet (2)
		Gel (3)

**Table 6:** System specification scores.

System attribute^1^	Scores				
	1	2	3	4	5
Bit resolution (bits)	14	16	22	24	>24
Sampling rate (Hz)	125 or 128	250 or 256	500 or 512	1000 or 1024	>1000
Battery life (hours)	Mains, USB or equivalent	1 to 8	9 to 16	17 to 24	>24

^1^A score of 1–5 is given separately for each system attribute and summed, along with the score for electrode type from Table 5, to give a single total score (minimum score = 4; maximum score = 20).

**Table 7:** Scoring of EEG system specification (*S*).

EEG system (published study)	Bit resolution (score)	Sampling rate Hz (score)	Battery life (score)	Electrode type^1^ (score)	Total system specification score (*S*)
ActiveTwo-1 (Davies and Gavin [20])	24 (4)	1024 (4)	10 (3)	Gel, active, shielded (5)	16
ActiveTwo-2 (Gramann et al. [7])	24 (4)	512 (3)	10 (3)	Gel, active, shielded (5)	15
ActiveTwo-3 (Gwin et al. [25])	24 (4)	512 (3)	10 (3)	Gel, active, shielded (5)	15
ActiveTwo-4 (Maidhof et al. [31])	24 (4)	8192 (5)	10 (3)	Gel, active, shielded (5)	17
actiCHamp (Bulea et al. [19])	24 (4)	500 (3)	24 (4)	Gel, active, shielded (5)	16
ANT-1 (Castermans et al. [4])	24 (4)	512 (3)	5 (2)	Gel, passive, shielded (4)	13
ANT-2 (Duvinage et al. [16])	24 (4)	2048 (5)	5 (2)	Gel, passive, shielded (4)	15
asalab (Ehinger et al. [10])	24 (4)	1024 (4)	10 (3)	Gel, active, shielded (5)	16
B-Alert (Robertson and Marino [32])	16 (2)	256 (2)	8 (2)	Cream, passive, shielded (4)	10
BrainAmp-1 (Jungnickel and Gramann [26])	16 (2)	500 (3)	30 (5)	Gel, active, shielded (5)	15
BrainAmp-2 (Wagner et al. [34])	16 (2)	2500 (5)	30 (5)	Gel, active, shielded (5)	17
BrainAmp-3 (Wascher et al. [2])	16 (2)	1000 (4)	30 (5)	Gel, active, shielded (5)	16
Cognionics (Lin et al. [29])	24 (4)	250 (2)	8 (2)	Dry, active, shielded (3)	11
EPOC-1 (Aspinall et al. [1])	14 (1)	128 (1)	6 (2)	Saline, passive, not stated (2)	6
EPOC-2 (Klonovs et al. [27])	14 (1)	128 (1)	6 (2)	Saline, passive, not stated (2)	6
EPOC-3 (Stopczynski et al. [33, 39])	14 (1)	128 (1)	6 (2)	Saline, passive, not stated (2)	6
MindWave-1 (Liu et al. [30])	16 (2)	500 (3)	10 (3)	Dry, passive, shielded (2)	10
MindWave-2 (Wong et al. [36])	16 (2)	500 (3)	10 (3)	Dry, passive, shielded (2)	10
Mobita (Askamp and van Putten [18])	24 (4)	2000 (5)	19 (4)	Gel, passive, shielded (4)	17
NuAmp (Wang et al. [35])	22 (3)	500 (3)	N/A (1)	Gel, passive, unshielded (3)	10
Oldenburg Hybrid-1 (Debener et al. [9])	14 (1)	128 (1)	6 (2)	Gel, passive, shielded (4)	8
Oldenburg Hybrid-2 (De Vos et al. [15])	14 (1)	128 (1)	6 (2)	Gel, passive, shielded (4)	8
Penso (Gargiulo et al. [24])	16 (2)	256 (2)	N/A (1)	Dry, passive, shielded (2)	7
Polymate AP216 (Lotte et al. [5])	16 (2)	1000 (4)	18 (4)	Gel, active, shielded (5)	15
Profusion (Fitzgerald et al. [23])	16 (2)	512 (3)	15 (3)	Gel, passive, unshielded (3)	11
SMARTING-1 (Debener et al. [21])	24 (4)	500 (3)	5 (2)	Gel, passive, not stated (3)	12
SMARTING-2 (Zink et al. [38])	24 (4)	500 (3)	5 (2)	Gel, passive, not stated (3)	12
Varioport (Doppelmayr et al. [22])	16 (2)	2000 (5)	96 (5)	Not stated, passive, not stated (1)	13
V-Amp (Zander et al. [37])	24 (4)	2000 (5)	N/A (1)	Dry, active, shielded (3)	13

^1^Selected from dry/saline/gel (or cream), passive/active, and unshielded/shielded.

**Table 8:** Overall scores for device mobility (*D*), participant mobility (*P*), system specification (*S*), number of channels (*C*), and total categorisation scores.

EEG system (published study)	Device mobility score (*D*)	Participant mobility score (*P*)	System specification score (*S*)	Number of channels (*C*)	Total categorisation score (*D*, *P*, *S*, *C*)
ActiveTwo-1 (Davies and Gavin [20])	0	0	16	32	*(0D, 0P, 16S, 32C)*
ActiveTwo-2 (Gramann et al. [7])	0	2	15	248	*(0D, 2P, 15S, 248C)*
ActiveTwo-3 (Gwin et al. [25])	0	4	15	248	*(0D, 4P, 15S, 248C)*
ActiveTwo-4 (Maidhof et al. [31])	0	1	17	32	*(0D, 1P, 17S, 32C)*
actiCHamp (Bulea et al. [19])	1	2	16	64	*(1D, 2P, 16S, 64C)*
ANT-1 (Castermans et al. [4])	0	2	13	32	*(0D, 2P, 13S, 32C)*
ANT-2 (Duvinage et al. [16])	0	2	15	128	*(0D, 2P, 15S, 128C)*
asalab (Ehinger et al. [10])	0	2	16	128	*(0D, 2P, 16S, 128C)*
B-Alert (Robertson and Marino [32])	3	2	10	20	*(3D, 2P, 10S, 20C)*
BrainAmp-1 (Jungnickel and Gramann [26])	1	1	15	156	*(1D, 1P, 15S, 156C)*
BrainAmp-2 (Wagner et al. [34])	0	2	17	120	*(0D, 2P, 17S, 120C)*
BrainAmp-3 (Wascher et al. [2])	1	4	16	28	*(1D, 4P, 16S, 28C)*
Cognionics (Lin et al. [29])	3	2	11	10	*(3D, 2P, 11S, 10C)*
EPOC-1 (Aspinall et al. [1])	3	3	6	14	*(3D, 3P, 6S, 14C)*
EPOC-2 (Klonovs et al. [27])	3	1	6	14	*(3D, 1P, 6S, 14C)*
EPOC-3 (Stopczynski et al. [33, 39])	4	0	6	14	*(4D, 0P, 6S, 14C)*
MindWave-1 (Liu et al. [30])	3	1	10	1	*(3D, 1P, 10S, 1C)*
MindWave-2 (Wong et al. [36])	3	1	10	1	*(3D, 1P, 10S, 1C)*
Mobita (Askamp and van Putten [18])	2	4	17	32	*(2D, 4P, 17S, 32C)*
NuAmp (Wang et al. [35])	3	1	10	32	*(3D, 1P, 10S, 32C)*
Oldenburg Hybrid-1 (Debener et al. [9])	3	3	8	14	*(3D, 3P, 8S, 14C)*
Oldenburg Hybrid-2 (De Vos et al. [15])	3	0	8	14	*(3D, 0P, 8S, 14C)*
Penso (Gargiulo et al. [24])	2	1	7	8	*(2D, 1P, 7S, 8C)*
Polymate AP216 (Lotte et al. [5])	1	3	15	3	*(1D, 3P, 15S, 3C)*
Profusion (Fitzgerald et al. [23])	2	1	11	32	*(2D, 1P, 11S, 32C)*
SMARTING-1 (Debener et al. [21])	4	0	12	16	*(4D, 0P, 12S, 16C)*
SMARTING-2 (Zink et al. [38])	3	3	12	24	*(3D, 3P, 12S, 24C)*
Varioport (Doppelmayr et al. [22])	2	3	13	10	*(2D, 3P, 13S, 10C)*
V-Amp (Zander et al. [37])	0	1	13	16	*(0D, 1P, 13S, 16C)*
