# Supplementary material for: Categorisation of Mobile EEG: A Researcher's Perspective
Source: Biomed Res Int. 2017 Dec 4;2017:5496196. doi: 10.1155/2017/5496196 (PMC5733835; doi:10.1155/2017/5496196)
Supplement: Supplementary file 1 — Categorisation of Mobile EEG (CoME) scoring form. [file 5496196.f1.pptx]

## Slide 1
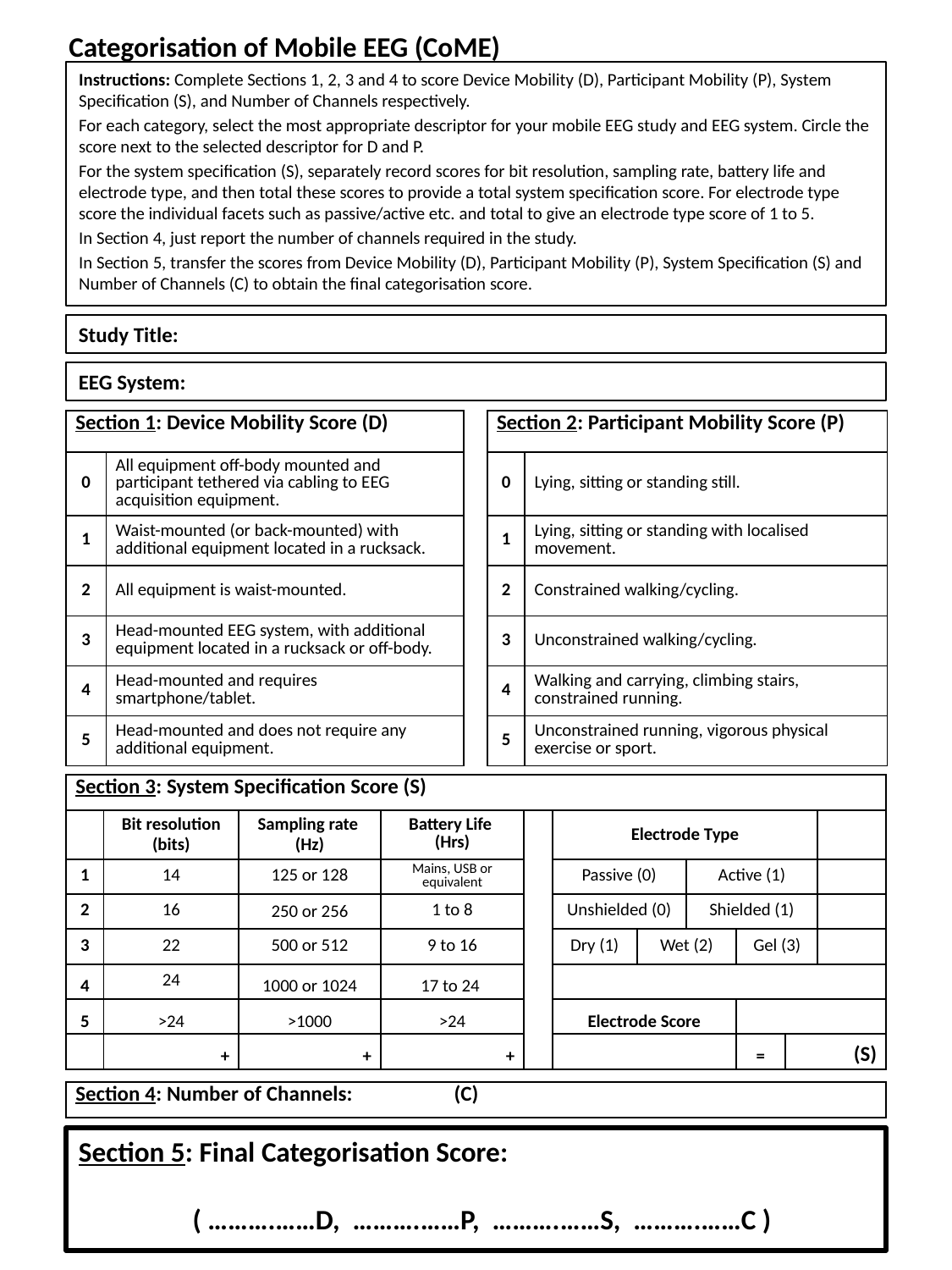

# Categorisation of Mobile EEG (CoME)
Instructions: Complete Sections 1, 2, 3 and 4 to score Device Mobility (D), Participant Mobility (P), System Specification (S), and Number of Channels respectively.
For each category, select the most appropriate descriptor for your mobile EEG study and EEG system. Circle the score next to the selected descriptor for D and P.
For the system specification (S), separately record scores for bit resolution, sampling rate, battery life and electrode type, and then total these scores to provide a total system specification score. For electrode type score the individual facets such as passive/active etc. and total to give an electrode type score of 1 to 5.
In Section 4, just report the number of channels required in the study.
In Section 5, transfer the scores from Device Mobility (D), Participant Mobility (P), System Specification (S) and Number of Channels (C) to obtain the final categorisation score.
Study Title:
EEG System:
| Section 1: Device Mobility Score (D) | | | Section 2: Participant Mobility Score (P) | |
| --- | --- | --- | --- | --- |
| 0 | All equipment off-body mounted and participant tethered via cabling to EEG acquisition equipment. | | 0 | Lying, sitting or standing still. |
| 1 | Waist-mounted (or back-mounted) with additional equipment located in a rucksack. | | 1 | Lying, sitting or standing with localised movement. |
| 2 | All equipment is waist-mounted. | | 2 | Constrained walking/cycling. |
| 3 | Head-mounted EEG system, with additional equipment located in a rucksack or off-body. | | 3 | Unconstrained walking/cycling. |
| 4 | Head-mounted and requires smartphone/tablet. | | 4 | Walking and carrying, climbing stairs, constrained running. |
| 5 | Head-mounted and does not require any additional equipment. | | 5 | Unconstrained running, vigorous physical exercise or sport. |
| Section 3: System Specification Score (S) | | | | | | | | | | |
| --- | --- | --- | --- | --- | --- | --- | --- | --- | --- | --- |
| | Bit resolution (bits) | Sampling rate (Hz) | Battery Life (Hrs) | | Electrode Type | | | | | |
| 1 | 14 | 125 or 128 | Mains, USB or equivalent | | Passive (0) | | Active (1) | | | |
| 2 | 16 | 250 or 256 | 1 to 8 | | Unshielded (0) | | Shielded (1) | | | |
| 3 | 22 | 500 or 512 | 9 to 16 | | Dry (1) | Wet (2) | | Gel (3) | | |
| 4 | 24 | 1000 or 1024 | 17 to 24 | | | | | | | |
| 5 | >24 | >1000 | >24 | | Electrode Score | | | | | |
| | + | + | + | | | | | = | (S) | |
| Section 4: Number of Channels: (C) |
| --- |
Section 5: Final Categorisation Score:
 ( ……….……D, ……….……P, ……….……S, ……….……C )
